# Influence of maize genotypes and harvest stages on in-silo fermentation quality and nutritional value of corn silage during hot summer condition of the tropics

**DOI:** 10.1186/s12870-024-05179-1

**Published:** 2024-06-03

**Authors:** Nadar Khan, Tawaf Ali Shah, Hafiz Muhammad Saleem Akhtar, Ahmad Mohammad Salamatullah, Mohammed Bourhia, Amare Bitew Mekonnen, Muhammad Zahoor Khan, Mudasir Nazar, Nazir Ahmad Khan

**Affiliations:** 1 Department of Animal Nutrition, The University of Agriculture, Peshawar, Khyber Pakhtunkhwa 25130 Pakistan; 2 Livestock and Dairy Development Department (Research), Peshawar, Khyber Pakhtunkhwa 25120 Pakistan; 3 College of Agriculture Engineering and Food Sciences, Shandong University of Technology, Zibo, China; 4 Department of Food Science & Nutrition, College of Food and Agricultural Sciences, King Saud University, 11 P.O. Box 2460, Riyadh, 11451 Saudi Arabia; 5 Laboratory of Biotechnology and Natural Resources Valorization, Faculty of Sciences, Ibn Zohr University, Agadir, 80060 Morocco; 6 College of Agronomy, Liaocheng University, Liaocheng, 252000 China; 7 Institute of Animal Science, Jiangsu Academy of Agricultural Science, Nanjing, 210014 China; 8 Department of Biology, Bahir Dar University, P.O.Box 79, Bahir Dar, Ethiopia

**Keywords:** Maize silage, Harvest maturity, Fermentation, Silage quality, Metabolizable energy, Carbohydrates subfractions

## Abstract

The aim of the experiment was to evaluate the potential of promising summer maize genotypes and optimal stage of harvesting these genotypes for ensiling in terms of dry matter (DM), starch, and crude protein (CP) yields, silage fermentation quality, nutrients profile, total digestible nutrients, metabolizable energy (ME) content, Cornell Net Carbohydrate and Protein System (CNCPS) carbohydrate (CHO) subfractions composition, in vitro DM digestibility (DMD) and in situ starch degradation characteristics. Six maize genotypes were chosen for the study: DK9108 from Monsanto, P30Y87, P3939 from Pioneer, QPM-300 (quality protein maize) and W94 from the International Maize and Wheat Improvement Center (CIMMYT), and a local cultivar, Afgoii, from the Cereal Research Institute (Persabaq, KP). A total of 72 plots (8 m × 10 m) were blocked in three replicate fields, and within each field, each genotype was sown in four replicate plots according to a randomized complete block design. For the data analysis, the Proc-Mixed procedure of Statistical Analysis System with repeated measure analysis of variance was used. The DM yield was strongly influenced (*P* < 0.001) by maize genotypes, varying from 12.6 to 17.0 tons/ha. Except for total CHO and ammonia nitrogen (NH_3_-N), the contents of all measured chemical components varied (*P* < 0.001) among the genotypes. Further comparison revealed that, genotype P3939 had a higher (*P* < 0.05) content of CP (7.27 vs. 6.92%), starch (36.7 vs. 27.9%), DMD (65.4 vs. 60.0%), ME (2.51 vs. 2.30 Mcal/kg) and lactic acid (5.32 vs. 4.83%) and lowest content of NDF (37.3 vs. 43.1%), pH (3.7 vs. 4.10) compared to the local cultivar (Afgoii). Advancement of post-flowering maturity from 25 to 35% DM (23 to 41 days after flowering (DAF)) increased (*P* < 0.05) the DM yield (10.4 to 17.8 tons/ha), starch content (29.1 to 35.0%), DMD (65.3 to 67.3%) and ME (2.34 to 2.47 Mcal/kg), and decreased (*P* < 0.001) the contents of CP (7.42–6.73%), NDF (48.8–38.5%), pH (4.10 to 3.60), NH_3_-N (8.93–7.80%N) and effective degradability of starch (95.4 to 89.4). Results showed that for higher yields and silage nutritional and fermentation quality, maize crops should be harvested at whole crop DM content of 30–35% (34 to 41 DAF). It was further concluded that genotype P3939 is the most suitable summer maize genotype for silage production in terms of yields and silage nutritional and fermentation quality under the hot environmental conditions of the tropics.

## Introduction

According to the Food and Agriculture Organization [1], the global demand for dairy products and food is projected to increase by 63% in 2050, with a significant portion of this increase originating from developing nations such as Pakistan due to their rising incomes and rapid population growth [2]. Pakistan is the fourth-largest milk producer in the world, producing more than 59,759 thousand tons of milk annually [3]. However, per-animal productivity is estimated to be less than one-third of their genetic potential [4]. A lack of high-quality forages, especially during prolonged periods of scarcity during winter and summer, is one of the primary causes of lower milk production in the country [5, 6]. The dairy industry of Pakistan is facing a profound challenge to meet the rising demand for milk, and a quest for an efficient, affordable, and sustainable increase in milk production is necessary [5]. It is only possible through production of sufficient quantities of good-quality forages, and preserving them for year-round availability [7].

Proper silage-making can preserve green fodder in optimum nutritional form for a long period of time [8]. Although silage-making technology was introduced into Pakistan a long time ago, there is still large room for optimizing ensiling practices and silage fermentation and nutritional quality, particularly by optimizing harvest maturity, forage genotypes and other agronomic management practices for local environment conditions. The quality of maize genotypes, harvest maturity stage, environment conditions (season and growth temperature), agronomic management are the key factors that can mainly modify maize silage yield, chemical composition and fermentation quality [8]. A meta-analysis revealed that the large variations in the chemical composition and nutritional quality of maize silages are mainly related to differences in maize genotypes and crop maturity at harvest [9, 10].

Harvest maturity, measured as days after flowering (DAF) or whole crop dry matter (DM) content, is the most important factor for quality silage production after the selection of a quality maize genotype, as it influences the biomass yield, fermentation quality, starch to neutral detergent fiber (NDF) ratio, NDF digestibility, and milk yield and composition of dairy cows [11, 12]. The crude protein (CP) content of the whole maize crop reduces with increasing maturity during the post-flowering period due to leaf senescence, while the contents of starch increase due to the accumulation of starch in the cob kernels [11]. On the other hand, the NDF content in the stover increases. However, the proportion of stover in whole-crop DM reduces due to a rapid increase in cob DM yield, which results in a decrease in NDF content in whole-crop DM during post-flowering maturity [12]. In addition, ensiling maize silage before its fully maturity stage can increase digestibility, but it also causes more DM and effluent losses and makes it less stable during in silo fermentation [13].

In Pakistan, maize is conventionally planted in summer season, specifically for the purpose of producing grain. The temperature can rise as high as 40–45 °C, and the day length reduces during post-flowering maturity, which can have an impact on the nutritional composition of maize silage [13, 14]. As far as we know, no study has been conducted to date on the effect of summer maize genotypes and post-harvest maturity stages on silage quality parameters. Therefore, this study was designed to screen out the best maize genotypes and determine the optimal stage of harvesting the maize crop for ensiling based on quantitative variables such as whole crop DM and DAF, by quantify changes in: yields of DM, starch, and CP; in silo fermentation characteristics; and nutrients profile, total digestible nutrients, metabolizable energy (ME) content, Cornell Net Carbohydrate and Protein System (CNCPS) carbohydrate (CHO) subfractions composition, in vitro DM digestibility (DMD) and in situ starch degradation characteristics, during post-flowering maturity under hot summer conditions.

## Materials and methods

### Experimental design and crop management

Six promising maize genotypes were chosen for the study, named: Dk9108 from Monsanto (Monsanto Co. Pvt. Pakistan), and P30Y87 and P3939 from Pioneer (Pioneer Hi-Bred International Inc., Pakistan). Quality Protein Maize QPM-300 and W94 from the International Maize and Wheat Improvement Centre (CIMMYT) Islamabad Pakistan, and Local Cultivar Afgoii from Cereal Research Institute Persabaq Khyber Pakhtunkhwa. The field trial was conducted in the agronomic research fields (34_020 North latitude, 71_480 East longitude, and 347 m above the sea level) of Peshawar Agriculture University, which has a semi-arid and subtropical climate. A total of 72 plots (8 m × 10 m) were blocked into three replicate fields, and within each field, each genotype was sown in four replicate plots according to a randomized complete block design. Maize seeds were sown manually with hands in ridges with a row-to-row spacing of 75 cm and plant-to-plant space of 20 cm at a seed rate of 66,000/ha. Standard agronomic, fertilization, irrigation, and weeds management practices were applied uniformly to all plots. Samples of the whole plant were harvested manually from a 1 m-long strip of two randomly selected adjacent rows of each plot at targeted DM contents of 25, 30, 35, and 40%, i.e., 23, 32, 41, and 48 days after flowering (DAF).

### Weather data

**Fig. 1:**
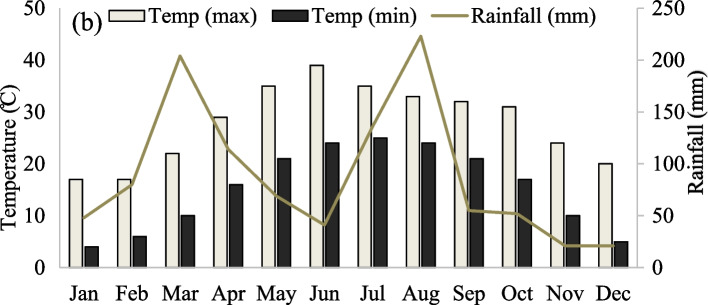
Total monthly rainfall (mm) and minimum and maximum temperature (°C) of the experimental area

The weather data record was received from the climate change centre, the University of Peshawar. Data regarding temperature and rainfall during the research experiment is given in Fig. 1 with a graphical presentation.

### Growth parameters, monitoring, maturity, and crop

The growth of maize crops was closely observed on a weekly basis by measuring the quantity of mature leaves. Additionally, the occurrence of flowering and silking, plant height, number of cobs per plant, and leaf senescence/plant were observed along a one-meter strip randomly picked two rows in each plot. Data on plant morphological characteristics were collected at three-day intervals following the emergence of silks. Additionally, to monitor the DM content of the whole crop, samples were collected at three-day interval after the appearance of silk in the crop.

### Sample collection and processing

The first cut was taken at a targeted whole crop DM content of 25% (19 DAF) to determine the biomass yield and chemical composition of maize. The next three cuts were taken at a targeted whole-crop DM content of 30% (27 DAF), 35% (34 DAF), and 40% (41 DAF). Samples of whole-crop maize were taken from a 1 m^2^ region of every plot during each harvest on a sunny day. A strip measuring 1 m in width was left unsampled on all sides of each plot, to avoid variation. Additionally, during successive harvests, a 1-m area next to the previously sampled area was excluded. The whole plant samples collected from each plot were labeled, covered with a cloth sheet to prevent exposure to light and direct air, and promptly transferred to the laboratory for data collection and subsequent processing. The number of plants harvested from each plot were accurately counted and weighed soon for fresh matter. After weighing, 10 plants of average size were collected, and the remaining whole crop sample from each plot was chopped at 0.5–1 cm. The 10 average size plants sampled from each plot were weighed, and divided into cob and stover fractions, each fraction was weighed, and subsequently chopped at 0.5–1 cm. Chopped samples of whole crop, stover, and cobs were immediately analyzed for DM content.

### Ensiling in laboratory silos

The chopped whole-crop maize was ensiled in laboratory silo with a capacity of 1.5 L. The silos were categorized based on the number of plots, genotype, date, and field, prior to being filled. The silage was added to the silos, layer by layer and compacted with steel rod. The lab silos were completely compressed and filled, and every possible measure to prevent the entrapment of oxygen in the bottle. After filling the lids were closed and sealed using a sealing tape, and ensiled for 90 days.

After 90 days, the silos were unsealed, and samples were collected for chemical composition and fermentation quality. The samples were air dried and ground to a particle size of 1 mm using a Wiley mill in the Animal Nutrition Laboratory. The contents of DM, CP, ash, ether extract (EE), acid detergent fiber (ADF) was assessed using the AOAC [15] methods. The NDF content was analyzed using the procedure outlined by Van Soest et al. [16]. The method described by Licitra et al. [17] was used to test the acid detergent-insoluble protein (ADICP) and the neutral detergent-insoluble protein (NDICP). The SCP content was determined by subtracting the CP content of residues from the total CP content. The total carbohydrate (CHO) content was estimated as CHO = 100 – EE – CP – ash [18].

### Silage fermentation quality

For assessment of the fermentation quality of maize silages, the subsamples, each weighing twenty grams, were immersed in 180 ml of water and left overnight at a temperature of 4 degrees Celsius. The solution was then passed through a double layer of nylon cloth using filtration. Subsequently, the filtrate was employed to measure the pH and quantify the ammonia nitrogen (NH_3_-N) and organic acids contents. The NH_3_-N was quantified by colorimetry using an auto-analyzer. The analysis of organic acids (lactic acid, acetic acid, and butyric acid) was conducted using high-performance liquid chromatography as previously described [19].

### Digestibility

For in vitro digestibility, the two-stage in vitro procedure of Tilley and Terry [20] was adopted for determining in vitro DM digestibility (DMD), as reported by Khan et al. [21].

### Digestible nutrients, energy values, and carbohydrate sub-fractions (CNCPS)

The carbohydrates subtractions were computed using the updated version of the Cornell Net Carbohydrate and Protein System (CNCPS; Higgs et al., and Van Amburgh et al. [22]). The carbohydrates were fractionated into CA1-subfraction (volatile fatty acids), CA2-subfraction (lactic acids), CA3-subfraction (organic acids), CA4-subfraction (soluble sugars), CB1-subfraction (starch), CB2-subfraction (soluble fiber), CB3-subfraction (available NDF), and CC-subfraction (unavailable NDF). The degradation rates (Kd) of the different subfractions in the rumen are: 0/h for CA1, 0.7/h for CA2, 0.5/h for CA3, 0.40–0.60/h for CA4, 0.20–0.40/h for CB1, 0.20–0.40/h for CB2, 0.04–0.09/h for CB3, and CC is non-degradable subfraction.

### Statistical analysis

The effects of summer maize genotypes and harvest maturity on the yields of DM, CP, and starch, the contents of DM, CP, Ash, EE, ADF, NDF, and starch, fermentation quality (pH, NH3-N, lactic acid, acetic acid, and propionic acid), in vitro DMD, CNCPS CHO subfraction, digestible nutrients, and energy values were determined by repeated measure analysis of variance using the PROC MIXED procedure of SAS (SAS Inst., Inc., Cary, NC). The harvest date was considered repeated effect on individual plots. Genotypes and harvest maturity were considered fixed effects, and replication was considered a random effect. We used the Akaike information criterion and the Schwarz Bayesian criterion, as explained by Littell et al. [23] and Wang and Gunawardena [24], for selection of covariance structure of repeated matrices. Means with different letters were obtained with "pdmix 800SAS macro".

## Results

### Growth and phenological characteristics of summer maize genotypes

Optimal growth and proper phenological characteristics of the maize plant are key factors for a high biomass yield and nutritive value for silage. The values of days to flowering (DTF; *P* < 0.001), days to silking (DTS; *P* < 0.001), plant height (*P* < 0.001), number of leaves per plant (NOL/P; *P* < 0.001), number of cobs per plant (NOC/P; *P* < 0.001), and leaf senescence per plant (LS/P; *P* < 0.01) varied among summer maize genotypes (Table 1). The highest DTF (71) and DTS (76) values from the day of sowing were observed for P3939, and the local maize cultivar Afgoii had the lowest values of DTF (64) and DTS (68). There was also large (*P* < 0.001) genetic variation in maize plant height, ranging from 193 cm (W94) to 235 cm (P3939). Similarly, the NOL/P varied (*P* < 0.001) among the summer maize genotypes, with the lowest value observed for W94 (13.6) and highest for P3939 (17.5). The LS/P differed (*P* < 0.001) among the summer maize genotypes, with Afgoii (5.80) and P3939 (3.70) showing the highest and lowest values, respectively.

**Table 1:** Growth and phenological characteristics of summer maize genotypes harvested at 34 days after flowering

Variety	Growth characteristics		Phenological parameters			
DTF	DTS	Height	NOL/P	LS/P		
Afgoii	64.0^c^	68.0^c^	220^c^	15.3^ cd^	5.80^a^	1.03^e^
W94	67.0^bc^	73.0^bc^	193^f^	13.6^e^	4.90^b^	1.10^de^
DK9108	69.0^b^	76.0^a^	213^d^	15.7^c^	4.00^c^	1.15^d^
P30Y87	69.0b	75.0^ab^	227^b^	16.0^b^	4.00^c^	1.33^c^
QPM300	70.0^ab^	74.0^b^	204^e^	14.8^d^	3.90^ cd^	1.48^b^
P3939	71.0^a^	76.0^a^	235^a^	17.5^a^	3.70^d^	1.67^a^
SEM	0.58	0.47	0.19	0.48	0.18	0.08
Significance	***	***	***	***	**	***

*DAF* day after flowering, *DM* dry matter, *DTF* days to flowering/tasselling, *DTS* days to silking, *NOL/P* number of leaves/plant, *LS/P* leaf senescence/plant, *NOC/P* number of cobs per plant

Mean with different superscription (abcd) within column differ at **P < 0.01 e *** *P* < 0.001

### Biomass and nutrients yield of summer maize genotypes

The yield (tons/ha) of DM, CP, NDF and starch showed large differences (*P* < 0.001) among the summer maize genotypes (Table 2). The P3939 had the highest yield (tons/ha) of DM (17), CP (1.17), starch (5.51) and NDF (7.31), while Afgoii had the lowest yield (tons/ha) of DM (12.6), CP (0.85), starch (3.51) and NDF (5.97).

**Table 2:** Biomass and nutrients yield of summer maize genotypes harvested at 34 DAF

Variety	DM (%)	Yield (tons/ha)				
		DM	CP	Starch	ADF	NDF
Afgoii	34.1^a^	12.6^c^	0.85^c^	3.51^e^	3.69^c^	5.97^d^
W94	33.8^ab^	14.6^b^	0.98^b^	4.39^d^	3.79^b^	6.58^b^
DK9108	33.4^b^	14.1^b^	1.02^b^	4.66^cd^	3.55^e^	6.42^c^
P30Y87	33.3^bc^	14.1^b^	0. 99^b^	4.89^c^	3.48^f^	5.96^d^
QPM300	32.8^cd^	15.0^b^	1.18^a^	5.28^b^	3.61^d^	6.58^b^
P3939	32.4^d^	17.0^a^	1.17^a^	5.51^a^	3.91^a^	7.31^a^
SE	0.20	0.24	0.02	0.09	0.21	0.13
Significance	***	***	***	***	***	***

*DM* dry matter, *CP* crude protein, *NDF* neutral detergent fibre, *ADF* acid detergent fibre

Mean with different superscription (abcd) within column differ at ***P < 0.001

### Chemical profile, in vitro dry matter digestibility and fermentation characteristics of summer maize genotypes

The results of the study revealed that, except for total carbohydrates (CHO), the contents of all measured chemical components varied (*P* < 0.001) among the genotypes. The highest (*P* < 0.05) content of CP was recorded for QPM300 (7.79% DM) and the lowest (*P* < 0.05) for the local cultivar Afgoii (6.92% DM). Moreover, QPM300 had the lowest (*P* < 0.05) content of soluble CP (SCP; 40.0% CP) and the highest (*P* < 0.05) content of ADICP (7.51% CP). Further comparisons revealed that P3939 had the highest content (*P* < 0.05) of starch, NFC, and in vitro DMD, and the lowest (*P* < 0.05) content of NDF and pH value (Table 3).

**Table 3:** Effect of maize genotypes on chemical composition and digestibility of whole crop silage harvested at 34 DAF

Genotypes	g/100 g dry matter						g/100 g CP			IVDMD (g/100 g)
	CP	CHO	Starch	ADF	NFC	NDF	SCP	NDICP	ADICP	
Afgoii	6.92^d^	84.0	27.9^e^	27.9^a^	40.0^c^	47.3^a^	40.9^b^	18.3^a^	7.50^ab^	60.0^f^
W94	7.12b^c^	85.0	30.1^d^	26.0^ab^	41.9^b^	45.1^ab^	42.4^ab^	17.0^b^	6.81^c^	62.1^e^
DK9108	7.19^b^	84.9	33.1^c^	25.5^bc^	41.3^bc^	45.6^ab^	42.6^a^	18.8^a^	7.22^abc^	62.9^d^
P30Y87	7.07^c^	85.4	34.7^b^	24.7^bc^	42.3^bc^	42.3^c^	41.9^ab^	16.2^c^	7.17^abc^	63.7^c^
QPM300	7.79^a^	84.5	35.8^ab^	24.1^cd^	42.1^bc^	43.9^b^	40.0^c^	18.6^a^	7.51^a^	64.6^b^
P3939	7.27^b^	85.5	36.7^a^	23.0^d^	44.7^a^	43.0^ac^	42.1^ab^	15.6^d^	6.87^bc^	65.4^a^
SEM	0.11	0.22	0.36	0.46	0.71	1.05	0.67	0.71	0.25	0.61
Significan	***	NS	***	***	**	***	*	**	**	***

*NFC* non-fibrous carbohydrates, *CHO* total carbohydrates, *SCP* Soluble CP, *NDICP* neutral detergent insoluble crude protein, *ADICP* Acid detergent insoluble crude protein, *DM* dry matter, *CP* crude protein, *NDF* neutral

Mean with different superscription (abcd) within column differ at *P < 0.05, **P < 0.01 and ***P < 0.001 detergent fibre, *ADF* acid detergent fibre, *IVDMD* in vitro dry matter digestibility

### Effect of summer maize genotypes on fermentation characteristics and carbohydrates CNCPS subfractions composition of whole crop maize silages

The fermentation parameters of six promising maize genotypes for maize silage are presented in Table 4. Our results demonstrated that P3939 contained a higher (*P* < 0.05) concentration of lactic acid and a lower pH than other maize genotypes. Silage produced from the W94 maize genotype contained higher (*P* < 0.05) acetic acid and pH. Moreover, there were no effect of maize genotypes on the concentration of total NH3-N. Additionally, after 90 days of fermentation, pH values ranged between 3.60 and 4.20, while no butyric acid was detected. Data on the effect of summer maize genotypes on the CNCPS carbohydrate subfraction composition of the silages are presented in Table. Except non-digestible CC subfraction, all other reported CNCPS subfractions varied (*P* < 0.05) among the genotypes. The P3939 had the highest (*P* < 0.05) value of rapidly degradable CA subfraction (17.6% DM) and intermediately degradable CB1 subfraction (36.7% DM), and lowest value of slowly degradable CB2 subfraction (40.9% DM). In contrast, Monsanto had the lowest (*P* < 0.05) values of rapidly degradable CA subfraction (12.2% DM) and local maize cultivar Afgoii has the lowest intermediately degradable CB1 subfraction (27.9% DM), and highest (*P* < 0.05) value of slowly degradable CB2 subfraction (52.7% DM).

**Table 4:** Fermentation parameters and CNCPS carbohydrate sub-fractions of whole crop maize silages produced from different genotypes harvested at 34 DAF

Genotypes	Fermentation parameters					CNCPS carbohydrate sub-fractions			
	pH	Lactic acid (%)	Acetic acid (%)	Propionic acid (%)	NH_3_-N (g/100 g N)	CA	CB1	CB2	CC
Afgoii	4.00^a^	4.83^b^	2.41^c^	1.0^c^	10.0	13.6c^b^	27.9^e^	52.7^a^	5.70
W94	4.10^a^	4.05^d^	2.89^a^	1.29^a^	9.70	14.1^b^	30.1^d^	50.9^b^	5.65
DK9108	3.90^ab^	4.65^bc^	2.61^b^	1.13^b^	9.60	12.2^c^	33.1^c^	49.7^ab^	5.30
P30Y87	3.80^ab^	4.43^c^	2.49^bc^	0.96^cd^	9.40	14.2^b^	34.7^b^	45.4^c^	5.60
QPM30	3.80^ab^	5.09^ab^	2.48^bc^	1.01^c^	9.20	16.1^ab^	35.8^ab^	43.0^d^	5.20
P3939	3.70^**b**^	5.32^a^	2.28^d^	0.91^d^	8.90	17.6^a^	36.7^a^	40.9^e^	4.90
SEM	0.31	0.32	0.13	0.09	0.43	0.75	0.66	1.58	0.29
Significance	***	**	*	*	NS	*	***	***	NS

*NH3-N* ammonia nitrogen, *CA* rapidly degradable (3.00/h) fraction, *CB1* intermediately degradable (0.20–0.50/h) fraction, *CB2* slowly degradable (0.02–0.10/h) fraction, *CC* unfermentable fraction, *NS* non-significant

Mean with different superscription (abcd) within column differ at *P < 0.05, **, *P* < 0.01, and ***P < 0.001

### Effect of summer maize genotypes on total digestible nutrients and estimated energy values

Data for total digestible nutrient (TDN) and estimated energy values of maize silages made from summer maize genotypes on are summarized in Table 5. Except tdFA, the values of all digestible nutrients (*P* < 0.05), TDN (*P* < 0.05) and estimated energy values (*P* < 0.01) of the silages differed among the evaluated genotypes. The P3939 had the highest (*P* < 0.05) values of tdNFC (43.9), TDN (71.9), DE (3.19 Mcal/kg) and ME (2.51 Mcal/kg), and lowest (*P* < 0.05) value of tdNDF (23.3). In contrast, local maize cultivar Afgoii had lowest (*P* < 0.05) values of tdNFC (40.5), TDN (66.9) and ME (2.30 Mcal/kg) and the highest (*P* < 0.05) value of tdNDF (25.3).

**Table 5:** Effect of maize genotypes on total digestible nutrients and estimated energy values of whole crop maize silage harvested at 34 DAF

Genotypes	tdNDF (%)	tdNFC (%)	tdCP (%)	tdFA (%)	TDN (%)	DE (Mcal/kg)	ME (Mcal/kg)
Afgoii	25.3^a^	40.5^c^	6.49^d^	2.02	66.9^c^	2.86^c^	2.30^c^
W94	24.8^ab^	41.5^b^	6.93^c^	2.18	69.7^bc^	3.00^bc^	2.38^bc^
DK9108	24.3^b^	40.9^bc^	6.95^c^	2.22	69.8^bc^	3.01^bc^	2.41^ab^
P30Y87	23.2^c^	41.9^ab^	7.00^b^	1.98	69.7^bc^	3.03^b^	2.41^ab^
QPM300	22.7^d^	42.5^ab^	7.70^a^	2.16	70.1^b^	3.08^a^	2.43^ab^
P3939	23.3^c^	43.9^a^	7.02^b^	2.12	71.9^a^	3.19^a^	2.51^a^
SEM	0.32	0.40	0.11	0.10	0.24	0.01	0.99
Significance	***	***	***	NS	*	*	*

*tdNDF* total digestible NDF, *tdNFC* total digestible non- fibre carbohydrates, *tdCP* total digestible CP, *tdFA* total digestible fat, *TDN* total digestible nutrients, *DE* digestible energy, *ME* metabolizable energy

Means with different superscription (abcd) within column differ at *P < 0.05 and ***P < 0.001

### Biomass yield, chemical profile, in vitro dry matter digestibility of the silages of maize ensiled at different post-flowering maturities

Changes in the yields (tons/ha) of DM, NDF, CP and starch of summer maize genotypes during post-flowering maturities are presented in Table 6. The DM yield increased from 11.7 to 18.5 tons/ha, from 19 DAF (24.4% DM) to 34 DAF (35.6% DM) stages of maturity. Similarly, the starch yield increased from 3.40 to 6.41 tons/ha, and NDF from 5.18 to 7.75 tons/ha, with an increase in harvest maturity from 19 to 34 DAF. In contrast, the yield of CP decreased from 1.12 tons/ha to 0.86 tons/ha with an increase in maturity from 19 to 41 DAF (24 – 35% DM).

**Table 6:** Changes in chemical composition, in vitro dry matter digestibility (DMD) of whole crop maize silages during post flowering maturity

Day after flowering (DAF)	DM (%)	DMY (tons/ha)	CP (%DM)	SCP (% CP)	NDICP (% CP)	ADICP (% CP)	NFC (%DM)	CHO (%DM)	Starch (%DM)	ADF (%DM)	NDF (%DM)	IVDMD (%DM)
19 DAF	24.4^d^	11.7^c^	7.34^a^	46.7^a^	09.9^c^	8.69^a^	36.4^d^	83.8	29.1^d^	29.3^a^	49.3^a^	65.3^d^
27 DAF	28.7^c^	14.2^b^	7.15^b^	43.7^b^	10.1^c^	6.05^b^	39.5^c^	84.6	32.3^c^	26.6^b^	47.1^b^	67.6^b^
34 DAF	35.6^b^	18.5^a^	7.04^c^	41.2^c^	14.4^b^	4.80^c^	44.3^b^	84.7	35.1^a^	24.8^c^	41.9^c^	68.8^a^
41 DAF	41.4^a^	18.2^a^	6.70^d^	40.2^d^	16.9^a^	3.50^d^	47.4^a^	85.6	35.0^a^	22.6^d^	39.8^d^	67.3^c^
SEM	0.60	2.80	0.09	0.25	0.37	0.18	0.33	0.17	0.30	0.33	0.31	0.15
Significance	***	***	***	***	***	***	***	NS	***	***	***	***

*DAF* days after flowering, *DM* dry matter, *DMY* dry matter yield, *NFC* non-fibrous carbohydrates, *CHO* total carbohydrates, *SCP* Soluble CP, *NDICP* neutral detergent insoluble crude protein, *ADICP* Acid detergent insoluble crude protein, *DM* dry matter, *CP* crude protein, *NDF* neutral detergent fibre, *ADF* acid detergent fibre, *IVDMD* Invitro dry matter digestibility

Mean with different superscription (abcd) within column differ at *P* < 0.05

Data on the effect of harvest maturity on proximate chemical composition, carbohydrates and CP chemical profiles, in vitro DMD and fermentation characteristics of silages are summarized in Table 6. Except for total carbohydrates (CHO), the content of all measured chemical components varied (*P* < 0.001) with harvest maturity. The highest (*P* < 0.05) content (7.34% DM) of CP was recorded at 19 DAF. The lowest (*P* < 0.05) content of CP (6.70% DM), SCP (40.2% CP) and highest (*P* < 0.05) content of NDICP (16.9% CP) was recorded at 41 DAF. Further comparison revealed that with advancing maturity from 19 to 41 DAF (24 – 40% whole crop DM), harvest stage, the contents of starch, NFC, and in vitro DMD increased (*P* < 0.05), whilst the pH value and NDF content decreased (*P* < 0.05).

### Fermentation quality

The effect of post-flowering harvest maturities on silage fermentation parameters is summarized in Table 7. The results demonstrated with increase in harvest maturity from 19 to 34 DAF, the lactic acid concentration increased (*p* < 0.05). The maximum lactic acid concentration was achieved at harvest maturity of 34 DAF. In contrast, pH, acetic acid and NH_3_-N concentrations decreased with increase in harvest maturity from 19 to 41 DAF.

**Table 7:** Fermentation Characteristics of whole crop maize silages production at different post-flowering maturity stages

DAF	DM%	pH	Lactic acid (% DM)	Acetic acid (% DM)	Propionic (% DM)	NH_3_-N (% N)
19	24.4^d^	4.10^a^	4.35^d^	2.95^a^	1.30^a^	9.20^a^
27	28.7^c^	3.90^b^	4.80^c^	2.60^b^	1.08^b^	8.70^b^
34	35.6^b^	3.80^b^	5.89^a^	2.23^c^	0.92^c^	8.30^c^
41	41.4^a^	3.60^c^	5.15^b^	2.00^d^	0.75^d^	7.90^d^
SEM	0.60	0.09	0.35	0.11	0.10	0.30
Significance	***	***	***	**	*	***

*DAF* days after flowering

Mean with different superscription (abcd) within column differ at *P < 0.05*, **P < 0.01, ***P < 0.001

### Carbohydrates CNCPS sub-fractions composition

Data on the effect of four different stages of post-flowering maturities on the CNCPS carbohydrate sub-fraction composition of the silages are presented in Table 8. All other reported CNCPS CHO sub-fractions varied (*P* < 0.001) at four post-flowering harvest maturities, except for the non-digestible CC sub-fraction. Advancing maturity from 19 to 41 DAF increased (*P* < 0.05) the values of rapidly degradable CA sub-fraction (15.2 to 17.4% DM) and intermediately degradable CB1 sub-fraction (29.1 to 35.7% DM). Whereas the value of slowly degradable CB2 sub-fraction) decreased from 52.1 to 42.0% DM.

**Table 8:** Effect of post flowering maturity on CNCPS carbohydrate sub-fraction of silages

Days after flowering (DAF)	Carbohydrate fraction			
	CA	CB1	CB2	CC
19 DAF	15.2^c^	29.1^d^	52.1^a^	3.61
27 DAF	13.8^d^	32.3^c^	50.2^b^	3.71
34 DAF	17.6^a^	34.6^b^	44.1^c^	3.64
41 DAF	17.4^b^	35.7^a^	42.0^d^	3.91
SEM	0.44	0.30	0.30	0.07
Significance	***	***	***	NS

a *CA*, rapidly degradable (3.00/h) fraction, *CB1* intermediately degradable (0.20–0.50/h) fraction, *CB2* slowly degradable (0.02–0.10/h) fraction, *CC* unfermentable fraction, *NS* non-significant

Mean with different superscription (abcd) within column differ at ***P < 0.001

### Total digestible nutrients and estimated energy values

Data on the effect of post-flowering maturity on digestible nutrients and estimated energy values of whole crop maize silages are summarized in Table 9. Except for tdFA, the values of all digestible nutrients (*P* < 0.001), TDN (*P* < 0.001) and estimated energy values (*P* < 0.001) of the silages varied due to post-flowering harvest maturity. The lowest (*P* < 0.05) values of tdNFC (35.6%), TDN (68.5%) and ME (2.34 Mcal/kg) and the highest (*P* < 0.001) value of tdNDF (28%) and tdCP (7.32%) were recorded at 19 DAF. In comparison, the highest (*P* < 0.05) values of tdNFC (46.7%), TDN (71.4%) and ME (2.47 Mcal/kg) and the lowest (*P* < 0.001) value of tdNDF (20.2%), tdCP (6.58%) were recorded at 41 DAF.

**Table 9:** Total digestible nutrients and estimated energy values of silages

Days after flowering (DAF)	tdNDF (%)	tdNFC (%)	tdCP (%)	tdFA (%)	TDN (%)	DE (Mcal/kg)	ME (Mcal/kg)
19 DAF	28.0^a^	35.6^d^	7.32^a^	2.00	68.5^c^	2.97^c^	2.34^c^
27 DAF	25.9^b^	38.6^c^	7.32^a^	2.18	69.6^b^	3.01^b^	2.40^b^
34 DAF	21.8^c^	43.7^b^	7.03^a^	2.24	70.7^a^	3.06^a^	2.44^ab^
41 DAF	20.2^d^	46.7^a^	6.58^b^	2.09	71.4^a^	3.08^a^	2.47^a^
SEM	0.26	0.33	0.09	0.08	0.20	0.01	0.04
Significance	***	***	**	NS	***	***	***

*tdNDF* total digestible NDF, *tdNFC* total digestible non- fibre carbohydrates, *tdCP* total digestible CP, *tdFA* total digestible fat, *TDN* total digestible nutrients, *DE* digestible energy, *ME* metabolizable energy

Means with different superscription (abcd) within column differ at **P < 0.01 and ***P < 0.001

#### Degradation kinetics of starch

The ruminal starch degradation kinetics and effective rumen degradability of maize silages at different harvest maturity stages are presented in Table 10. Our findings showed large differences in soluble (W; *p* < 0.01), potentially rumen-degradable (D; *p* < 0.01), and rumen-undegradable fraction (U; *p* < 0.05), rate of degradation (Kd; *p* < 0.05) effectively rumen-degradable starch (EDstarch; *p* < 0.05), and undegradable starch (UDstarch; *p* < 0.05). With the advancing of harvest maturity from 19 to 34 DAF, the values of W, Kd, and EDstarch increased (*p* < 0.05), whereas the maximum (*p* < 0.05) values of D, U, and UDstarch were observed when the crop was ensiled at 41 DAF of harvest maturity, as compared to early stages.

**Table 10:** Effect of harvest maturity on ruminal starch degradation kinetics and effective rumen degradability of maize silages

Day after flowering (DAF)	Rumen degradation characteristics				EDstarch	UDstarch
	W	D	U	kd		
19 DAF	31.2^d^	54.8^ab^	2.65^d^	18.5^b^	89.4^b^	13.3^b^
27 DAF	35.4^c^	52.1^b^	3.10^c^	21.1^ab^	87.6^d^	14.7^ab^
34 DAF	44.1^a^	57.6^a^	4.45^b^	23.2^a^	94.8^a^	11.8^c^
41 DAF	39.3^b^	45.6^c^	5.15^a^	16.8^c^	91.4^b^	15.3^a^
SEM	1.34	1.06	0.80	0.45	2.52	1.5
Significance	**	**	*	*	*	**

*SEM* standard error of the mean, *W* washable or soluble fraction, *D* potentially rumen degradable fraction, *U* undegradable fraction, *Kd* rate of degradation, *EDstarch* effective rumen degradable starch, *UDDM* undegraded starch, *DAF* Days after flowering

Means with different superscripts (abc) within the columns differ at *P < 0.05and **P < 0.01

## Discussion

The high tropical summer temperature during post-flowering maturity and ensiling, strongly influence the epiphytic microbial population, in-silo fermentation characteristics and nutritional value of maize silage [12]. The temperature during summer in Pakistan often exceed 40 °C. Silage production under such hot conditions, particularly in the face of global warming has been envisaged as a major challenge for future silage production, particularly in the tropic. This study presents the first comprehensive dataset on silage production from six promising summer genotypes, harvested at four maturities stages during tropical summer conditions, where temperature can exceed 40 °C. The comprehensive dataset generated in this study include data on the biomass, starch and CP yields, silage fermentation quality, nutrient profile, CNCPS CHO subfractions, DMD (in vitro), TDN, ME and in situ starch degradation characteristics under the hot summer conditions of the tropics. Furthermore, the nutrient composition changes during maturity were marked by easily measurable quantitative variables such as whole crop DM content and DAF.

Maize phenology is very important for the determination of the main components (leaf, stem, and cob) of whole crop maize plants and can vary among the genotypes and growing conditions. The genotypic variation in growth and phenological characteristics of maize plants affects the biomass yield, fermentation quality, and nutrition value of the silages. In the current study, there was a large variation among the summer maize genotypes in growth rate and phenological characteristics. The DTS of the maize genotypes in our study varied from 57 (Afgoii) to 76 (Pioneer). In agreement with our findings, the DTS in commercial maize hybrids varied from 68 to 76 [25]. The variation in genotypes may be due to their genetic makeup, early and late germination, nitrogen uptake capacity, adaptation to a certain soil, and maturation period. Plant height is an important component of maize growth and reflects plant growth attained during the growing period, as well as the nutrient quality and quantity of the whole maize crop. In the current study, the plant height ranged from 193 to 235 cm among the summer genotypes, which is consistent with the values (190 to 250 cm) reported in earlier studies [26]. The values for NL/P and NC/P in the maize crop reflect the yield, nutrient content, and silage quality. The number of green leaves is closely associated with plant height, digestibility, and DM yield of the crop. In the current study, the NC/P ranged from 1.03 to 1.67 and the NL/P ranged from 13.6 to 17.5 for different summer maize genotypes. Our results are in line with the earlier reported values for NC/P (0.90 to 1.61) and NL/P (14 to 18) [25]. This variation in plant height, leaf size, and cobs per plant may be due to variation in their genetic makeup such as the maturity stage of these maize genotypes.

Selecting suitable maize genotypes is key for achieving high forage yields and nutritional quality [27]. The nutrient contents and yield of the key nutrients (CP, starch, and NDF) significantly varied within maize genotypes. The range in CP content (6.92 to 7.79% DM) is consistent with earlier findings, which reported that CP of maize silage varied from 6.9 to 8.10% DM among maize genotypes [28]. The differences in CP content may be due to variations in leaf number per plant, leaf senescence, stover to cob ratio, and maturity. The content of starch in silages was highly variable (27.9 to 36.7%) among the maize genotypes, which were consistent the range of starch values (23.0 to 35.5%) reported in the literature review [11]. The NDF content ranged from 42.3 to 47.3%, which is in line with earlier reported NDF values of 37.5 to 51.1% [29]. The differences in starch and NDF content may be due to variations in stover to cob ratio, kernel fraction, and time for starch content of kernels.

A proper harvest stage at post-flowering maturity is important for silage quality and biomass yield. In the current study, the DM yield increased from 11.7 to 18.5 tons/ha with an increase in maturity from 19 DAF (25% DM) to 34 DAF (35% DM). The results are consistent with earlier reported values (13.2 to 17.1 tons/ha) with an increase in the maturity stage of whole crop DM content of 29.6 to 42.1% [9, 33]. This variation in DM yield may be due to grain filling and an increase in grain and cob mass with increasing harvest maturity during the post-flowering period [10]. As maturity progressed from 19 to 41 DAF, the CP yield decreased from 1.12 to 0.82 tons/ha. Literature data show that the CP yield and content may be reduced by 3% with advancing maturity from 25 to 45% DM [33]. The decrease in CP content may be due to decreased leaf fraction in stover DM, decreased leaf-to-stem ratio, and increased leaf senescence [7, 9]. The increase in starch yields from 3.40 to 6.40 ton/ha with advancing maturity from 19 to 41 DAF was consistent with the findings of earlier studies [9]. This increase in the starch yield with post-flowering maturity may be due to a rapid grain filling during the post-flowering period, an increase in kernel fraction, and the advancing maturity stage, which provides more time for starch deposition and a gain in grain mass.

From the start of flowering until full maturity, very rapid changes occur in various morphological fractions of the plant. The cob undergoes several stages of physiological changes before full maturity, which are reflected by the increase in the content of starch and grain mass of the whole plant. In the current study, the content of CP decreased from 7.34 to 6.70%, with an increase in DAF from 19 to 41. In agreement with our findings, the CP values decreased from 8.6 to 6.9% with an increasing maturity from 25 to 40% DM [34]. This is probably due to the fact that leaf senescence increases as maturity progresses, thus reducing the green leaves and CP content [29, 35]. There was a large decrease in the content of SCP and increase in NDICP with advancing maturity from 19 to 41 DAF. The current results align with the study of Higgs et al. [36], who recorded that the concentration of SCP decreased and NDICP increased with increasing post-flowering maturity of maize crop. In the current research, the starch content increased from 29.1 to 35.1%, with advancing maturity from 19 to 34 DAF. Our results are consistent with the values (23 to 39%) for increase in starch content reported in literature [9, 37]. The increase in starch content can be related to the rapid grain filling during advancing post-flowering maturity, a high kernel fraction, and the maximum time for starch deposition. In the current study, the content of NDF decreased from 49.8 to 39.3% with advancing maturity from 19 to 34 DAF, which is consistent with the earlier reported values (54.5 to 37.7%) [34]. This decrease in NDF content with the advancing maturity of maize silages was associated with increased starch and grain filling in the crop plant. This increase in the grain proportion of the plant dilutes the fibrous portion, reducing the content of NDF [38].

A pH of less than 4.0 is a good indicator of the fermentation quality of maize silages. The pH ranged from 3.60 to 4.20 in silages made from summer genotypes and was consistent with reported values of pH ranging from 3.74 to 4.06 [31]. The homofermentive lactic bacteria produce different concentrations of lactic acid during the fermentation of silage, which is vital, as abundant organic acids are needed during the ideal fermentation. Our results were within the recommended range of 4–6%, similar to those of Nennich et al. [32], suggesting the maize silage was well fermented in this experiment. Additionally, the NH_3_-N concentration in maize silages reflects the level of proteolysis and indicates the degradation of CP. Reported values of NH_3_-N ranged from 7.54 to 9.83% of total N. Earlier studies reported that the NH_3_-N content should not exceed more than 10% of total N for quality silages [21]. Butyric acid was not detected in this experiment. Generally, butyric acid concentration was low and biologically negligible in whole-plant maize silage [12]. In the current study, the pH decreased from 4.10 to 3.60 with advancing maturity from 19 to 41 DAF. The reduction in pH during the ensiling process needs a high amount of water-soluble carbohydrates for lactic acid production. For proper fermentation and quality silage production, early pH reduction and rapid accumulation of lactic acid in silage prevent the growth of anaerobic bacteria and mould that cause spoilage. This also helps minimize the nutrient loss from the silage caused by plant enzymes and anaerobic microbes, with less proteolysis occurring. Advancing plant maturity tends to reduce NDF content and increase starch content, determining the VFA production profile, namely, increasing lactic acid and decreasing acetate production [34]. With further maturity, the content of water-soluble carbohydrates in maize crops decreases. NH_3_-N is a good indicator of silage fermentation quality. A high value indicates extensive protein degradation (amino acids) through proteolysis. In the current study, the NH_3_-N content varied and decreased from 9.2 to 7.9% total N with advancing harvest maturity from 19 to 41 DAF (25–40% DM), which is consistent with literature values [37].

For high DM intake and milk production, the digestibility of the whole crop of maize silages is very important. The variation in DMD ranged from 60.0 to 65.4% in maize silages made from different summer genotypes. The results are in accordance with reported values of DMD, ranging from 61.7 to 65.0% [11, 30]. High digestible forage in the ration is important for high DM intake and milk production [39]. In the current study, the DMD increased from 65.3 to 68.8%, with increasing maturity from 19 (25% DM) to 34 DAF (35.0% DM). Our results are consistent with literature values, which reveal that the DMD of maize silages increases from 2 to 4% with the advancing stage of maturity, from 24 to 38% DM [37]. This increase in digestibility with maturity can be related to the increase in the starch content and decrease in NDF content in the whole crop, which increased the digestibility of the whole crop, despite a decrease in digestibility of the stover fraction during post-flowering maturity [40].

The CNCPS is widely used for carbohydrates and protein nutritional value evaluation in feedstuffs for ruminants and for diet formulation according to dairy cattle requirements [41, 42]. Advancing the maturity from 19 to 41 DAF increased the value of the intermediately degradable CB1 sub-fraction and decreased the slowly degradable CB2 sub-fraction. This might be due to the accumulation of starch and decrease in NDF content with advancing maturity. Our results are in line with Gupta et al. [43] and Refat et al. [44]. Advancing the harvest maturity stages from DAF 19 to 34 resulted in a significant increase in W, Kd, and EDstarch values. This indicates that as the maturity stage progressed, there was an increase in the weight, kernel density, and starch energy density. On the other hand, the maximum values of D, U, and UD starch were attained at DAF 41 of harvest maturity when compared to the earlier stages. This suggests that 41 DAF (39.3% DM) is the optimal maturity stage for achieving the highest values for kernel diameter, kernel hardness, and starch hardness. These findings highlight the importance of considering the harvest maturity stage when assessing these parameters, as they show distinct variations throughout the maturation process [45, 46].

Except for tdFA, all digestible nutrients, TDN, and estimated energy values of the silages varied during post-flowering maturation. With advancing maturity from 19 to 34 DAF, the value of TDN increased from 68.5 to 71.4% and ME from 2.34 to 2.47 Mcal/kg. Our results were consistent with the literature values of Nazli et al. [30]. The increase in energy values may be due to the maximum time for deposition of starch and rapid grain filling, with a decrease in the NDF content of the whole maize plant [47].

## Conclusions

The In conclusion, the selection of a suitable maize genotype and optimal post-flowering harvest maturity significantly impacts the biomass and nutrient yields, as well as the digestibility and silage fermentation quality. Compared to the local cultivar Afgoii, genotype P3939 performed with a higher DM yield (17.0 vs. 12.6%), content of CP (7.27 vs. 6.92%), starch (36.7 vs. 27.9%), DMD (65.4 vs. 60.0%), ME (2.51 vs. 2.30 Mcal/kg) and lactic acid (5.32 vs. 4.83%) and lowest content of NDF (37.3 vs. 43.1%) and pH (3.7 vs. 4.10). Harvesting maize at 34 to 41 days after flowering (DAF) or whole crop DM of 30–35% resulted in increases in DM yield (10.4 to 17.8 tons/ha) and starch (2.87 to 6.17). The findings of the study suggest that harvesting the whole maize crop at 34 to 41 DAF (DM 30–35%) optimizes biomass and nutrient yields and enhances the silage quality and fermentation process. Overall, among the genotypes evaluated, P3939 emerges as the most suitable summer maize genotype for silage production in the hot climatic conditions of Pakistan.

## Data Availability

All data generated or analyses during this study are included in this published article.
